# Can a Resident's Publication Record Predict Fellowship Publications?

**DOI:** 10.1371/journal.pone.0090140

**Published:** 2014-03-21

**Authors:** Vinay Prasad, Jason Rho, Senthil Selvaraj, Mike Cheung, Andrae Vandross, Nancy Ho

**Affiliations:** 1 Medical Oncology Branch, National Cancer Institute, National Institutes of Health, Bethesda, Maryland, United States of America; 2 Northwestern University, Department of Medicine, Chicago, Illinois, United States of America; 3 Brigham and Women's Hospital, Department of Medicine, Boston, Massachusetts, United States of America; 4 Yale University, Department of Medicine, New Haven, Connecticut, United States of America; 5 University of Maryland, Department of Medicine, Baltimore, Maryland, United States of America; Banner Alzheimer's Institute, United States of America

## Abstract

**Background:**

Internal medicine fellowship programs have an incentive to select fellows who will ultimately publish. Whether an applicant's publication record predicts long term publishing remains unknown.

**Methods:**

Using records of fellowship bound internal medicine residents, we analyzed whether publications at time of fellowship application predict publications more than 3 years (2 years into fellowship) and up to 7 years after fellowship match. We calculate the sensitivity, specificity, positive and negative predictive values and likelihood ratios for every cutoff number of application publications, and plot a receiver operator characteristic curve of this test.

**Results:**

Of 307 fellowship bound residents, 126 (41%) published at least one article 3 to 7 years after matching, and 181 (59%) of residents do not publish in this time period. The area under the receiver operator characteristic curve is 0.59. No cutoff value for application publications possessed adequate test characteristics.

**Conclusion:**

The number of publications an applicant has at time of fellowship application is a poor predictor of who publishes in the long term. These findings do not validate the practice of using application publications as a tool for selecting fellows.

## Introduction

Fellows in the subspecialties of internal medicine must demonstrate evidence of research productivity, including publications, in accordance with current Accreditation Council for Graduate Medical Education guidelines [1] As such, fellowship program directors have incentive to select applicants who are likely to engage in scholarship and publish those results. One criterion used by fellowship programs to predict who will publish during and beyond fellowship is the number of publications a resident has at the time of application. The logic here is straightforward. Applicants, who publish more prior to fellowship, are likely to publish more in the long term. While no data exists for fellowship programs, one group has found that medical students with stronger publication records were ranked more favorably by general surgery residencies [2].

Although the idea that past publications predict future ones is accepted informally, and widely used to advise fellowship applicants, it has rarely been examined in peer review literature, and there is little published evidence in support of this claim. In part, this may be because fellowship selection procedures are guarded at the institutional level. One investigation of the publication habits of urology residents concluded there was a strong positive correlation between publications during residency and those afterwards (Spearman's rank correlation = 0.5, p<0.001) [3].

However, simply showing a statistically significant relationship between early publications and future publications does not mean that this fact is meaningful, or that it can be used to better select applicants. The key question is whether correlations offer ability to predict future outcomes. To understand this concept consider that fellowship directors must use all of the information applicants select to make a decision as to who will be interviewed. Some of the variables that program directors consider are: USMLE scores, medical school, hospital of residency, history or research, and the essay [4]. However, another consideration is past research projects and interests, of which publications serves as a measure. Even if an applicant's publications are only used as an adjunct factor to selecting those to interview (as is the case), publications are discrete variables (applicants have either 0, 1, 2, or *n* many papers). Thus, one would be interested to know whether or not any discrete number serves as a useful cut-point. This is analogous to knowing that certain d-dimer level excludes pulmonary embolism, rather than merely knowing that risk increases with rising numbers. To best assess whether application publications can predict future publication one can construct a receiver-operator-characteristic curve to see whether any cut point offers reliable test characteristics. This point has been made elsewhere, regarding medical education publications [5] [6], and medical tests [7].

Here, we sought to investigate formally whether publications at time of fellowship application predict future publications in a sample of fellowship bound internal medicine residents. We report the sensitivity, specificity, positive and negative predictive values (PPV and NPV) of application publications as predictor of future publications, and present a receiver operator characteristic (ROC) curve for this test.

## Methods

We assembled a database of fellowship bound internal medicine residents and their publication histories, described fully elsewhere [8] and summarized here. Using an Internet search engine (Google), we obtained publically available records of internal medicine residency fellowship match results. As all data we acquired was in the public domain, our investigation did not require institutional review board approval.

In contrast with other graduate fields and medical specialties, there is no published ranking for internal medicine residency programs. Programs were searched if they were associated with the top 20 medical schools according to US News and World Report Graduate School Rankings 2013. The following residency programs were searched: Brigham and Women's Hospital, Massachusetts General Hospital, Beth Israel Deaconess Medical Center, Johns Hopkins University Medical Center, Columbia University Medical Center, Yale University Medical Center, The University of Pennsylvania Medical Center, Washington University Medical Center in St. Louis, University of Washington Medical Center, Duke University Medical Center, Cornell Medical Center, University of Michigan Medical Center, Northwestern Memorial Hospital, University of Chicago Medical Center, Mount Sinai Medical Center, University of California San Francisco Medical Center, University of California Los Angeles Medical Center, University of California San Diego Medical Center, University of Texas Southwestern Medical Center, Stanford University Medical Center, University of Pittsburgh Medical Center, and Vanderbilt University Medical Center.

Programs were eligible if they listed the fellowship placement of a resident by name, location, and specialty. Data from centers were excluded if lists of resident placement were not published by year, if published resident placement list sizes varied significantly from year to year, raising the question of whether publish lists were complete, and/or if data for residents who pursued general medicine or hospital medicine was not listed.

Data was obtained for the following programs and the following match years: Columbia (2007–2009), University of Chicago (2006–2009), University of California Los Angeles (2006–2009), University of California San Diego (2005–09), University of Pennsylvania (2008–09). Residents who did not pursue fellowship were excluded from this analysis. Residents were considered fellowship bound if they pursued one of the following specialties: hematology/oncology, pulmonary, critical care, pulmonary/critical care, endocrinology and metabolism, gastroenterology, cardiovascular disease, nephrology, rheumatology, geriatrics, and infectious disease.

### Publication histories

Data on the publication record of each resident were obtained by PubMed query using multiple search strategies, including, but not limited to 1) full name 2) last name plus first initial and residency program 3) last name plus first initial and fellowship program, 4) last name plus first initial and field of interest. When a resident was identified, his/her PubMed record was explored both backwards and forwards in time, using additional search strategies, including, but not limited to, unique initials, coauthors, subject matter, and principal investigators. Data was de-identified after publication history was obtained for analysis datasets.

The publications of internal medicine graduates were considered in two-year intervals. The fellowship match (defined as June 15^th^ of the corresponding year) was used to align all records and defined as the zero point in time. One year before and after this date was termed the ‘perimatch’ period, and labeled accordingly on all graphs. Publications during the ‘perimatch’ period were considered among the publications at time of application, though some occurred up to one year after matching. As the Electronic Residency Application Service (ERAS) service allows applicants to report publications under review at a journal, we feel that a one-year inclusion post match approximates an applicant's submitted publication record on the ERAS application.

When graduates only completed a portion of their final two-year interval (for instance 18 out of 24 months), their rate of publication was assumed to be constant for that interval, and adjusted accordingly. We examined the data of 307 residents with follow up at least 3 years after fellowship match or more than 2 years after the start of fellowship, and up to 7 years after fellowship match. The full data set from which this project was derived had a robust rate of data acquisition. Data was obtained for 823 out of 922 names, a recovery rate of 89.3%. We were unable to recover data in cases where a search strategies returned a large number of results (e.g. John Smith, Neha Patel) and results could not be meaningfully filtered by institutional affiliation. Thus, accurate delineation of publications became unreasonable, and the name was omitted.

### Statistical analysis

Students' t-test was used to compare the mean publications between late publishers and non-publishers. The formal test characteristics of application publication records were calculated as described above.

## Results

**Figure 1:**
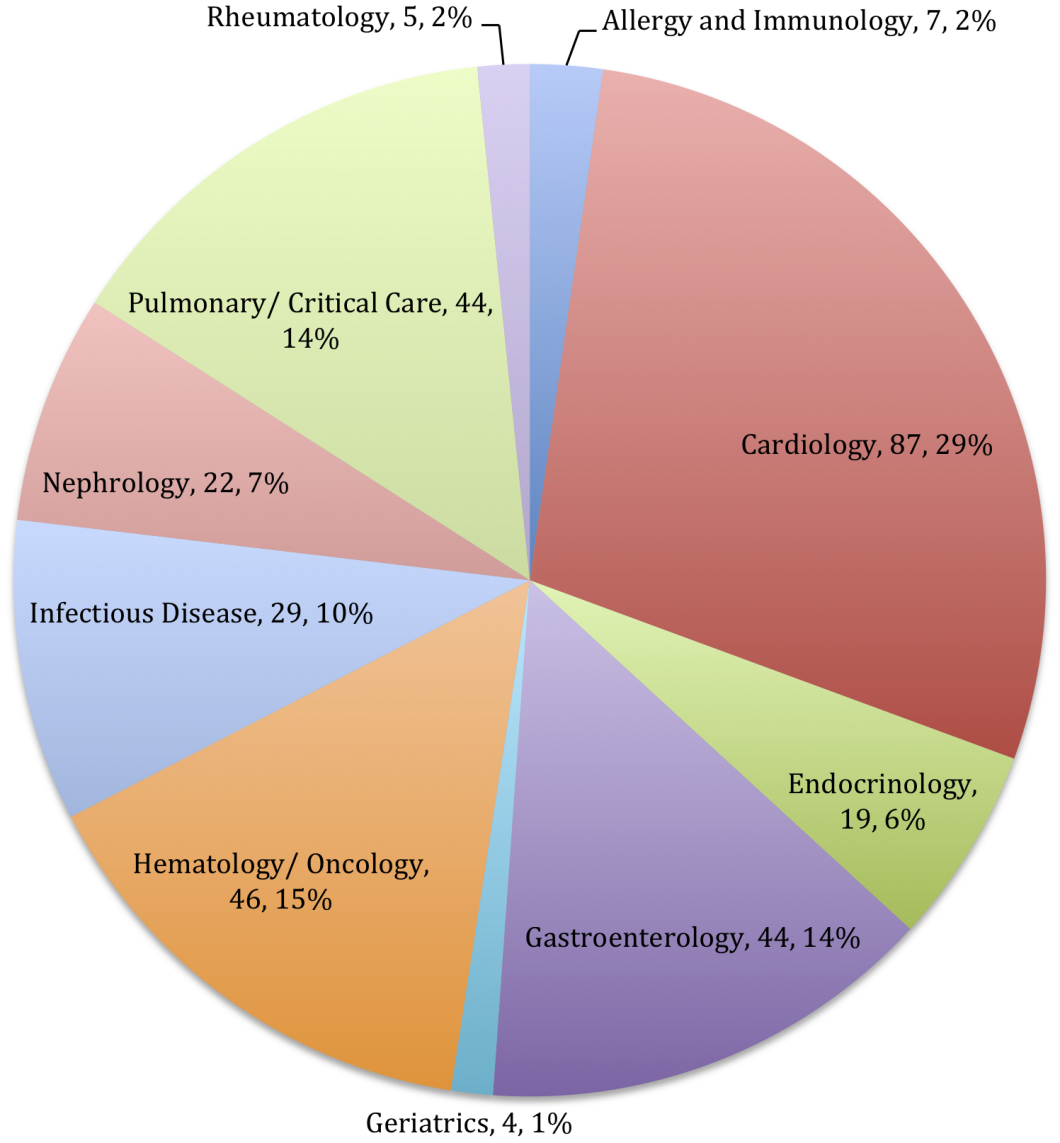
Provides a breakdown of specialty choice among this group of internal medicine graduates.

**Figure 2:**
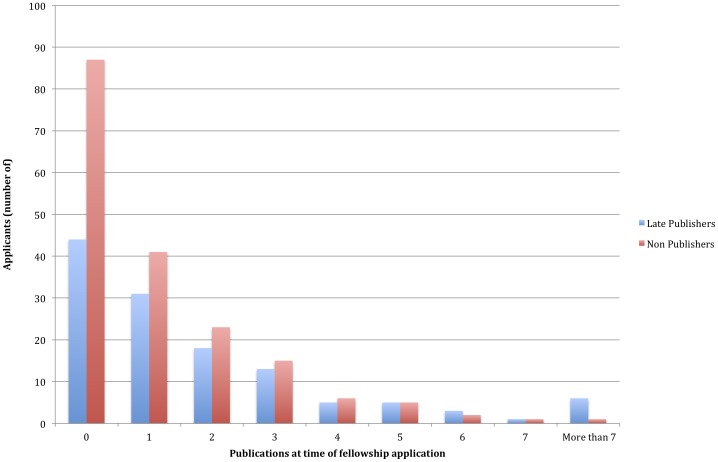
Shows the relationship between the number of publications at time of fellowship application, stratified by which applicants went on to publish (Late Publishers) or who never subsequently published (Non-Publishers).

Our dataset, fully described elsewhere [8], included 307 residents for whom we had documented follow up of at least 2 years after the start of fellowship, and up to 7 years after fellowship match. Of these 307 fellowship bound internal medicine residents, 126 (41%) ultimately published at least one article between 3 and 7 years after fellowship match (Late Publishers). The remainder, 181 residents (59%) did not publish during this time (Non Publishers). The specialty choices of these residents are varied, and are shown in **Figure 1**. The number of publications at time of fellowship match for both Late and Non Publishers are shown in **Table 1**. Of note, the majority of fellows publish between 0 to 3 articles during the study period. **Figure 2** shows the number of manuscripts fellows had published at the time of fellowship application, stratified by whether they published at least 1 manuscript (Late Publishers) after the match. Late Publishers had more manuscripts at time of application than non-publishers (2.03 vs 1.19, p = 0.008)

**Table 1:** A breakdown of fellowship applicants by the number of publications at time of application, among those who published in the future (late publishers) and those who did not (non-publishers).

Articles at time of fellowship application	Late Publishers	Non Publishers
0	44	87
1	31	41
2	18	23
3	13	15
4	5	6
5	5	5
6	3	2
7	1	1
More than 7	6	1

**Figure 3:**
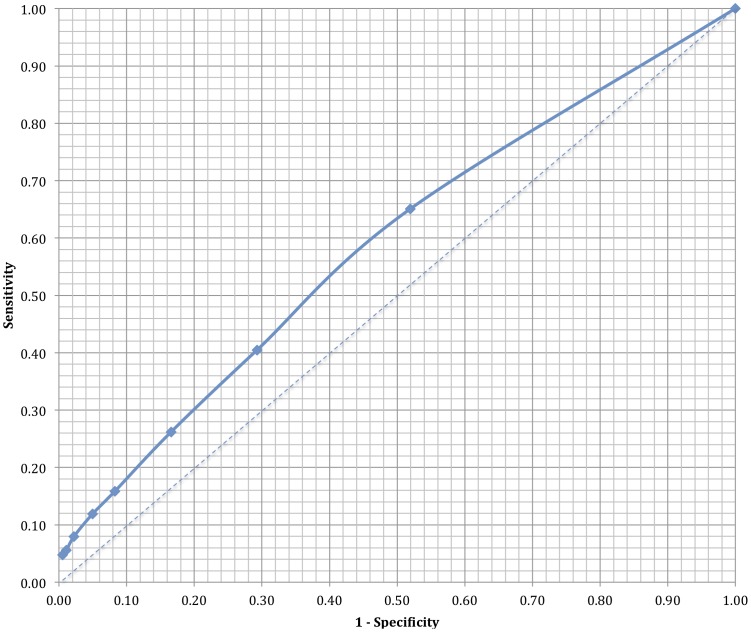
Depicts a receiver operator characteristic curve for publications at time of fellowship application as a predictor of future publication.

**Figure 4:**
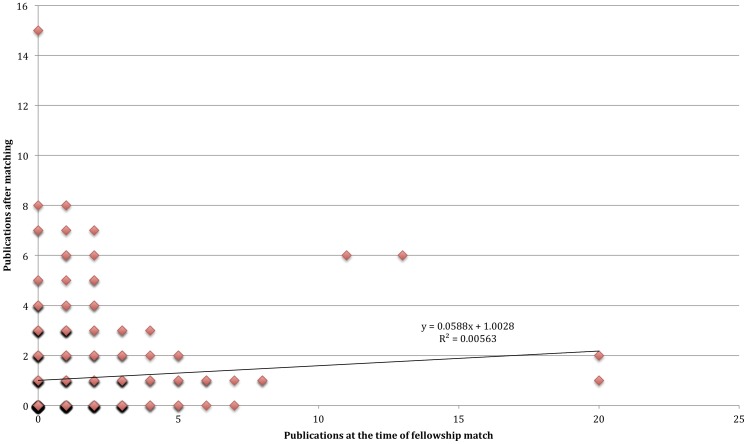
Shows the relationship between publications at time of fellowship application and future publications.

The sensitivity, specificity, positive and negative predictive values and likelihood ratios at various cut-off values for the number of publications at time of match is shown in **Table 2**. A receiver operator characteristic curve was constructed (**Figure 3**), with an area under the curve noted to be 0.59. **Figure 4** shows a simple scatterplot of publications at the time of fellowship application (x-axis) versus those that occurred thereafter (y-axis). The r-squared coefficient for a linear regression of the data is 0.005.

**Table 2:** The sensitivity, specificity, and positive and negative likelihood ratios for all cutoff values of publications at time of application as a predictor of future publication status.

Cutoff	Sensitivity	Specificity	PPV	NPV	+LR′	−LR′
0	100.0	0.0	41.0		1.00	
1	65.1	48.1	46.6	66.4	1.25	0.73
2	40.5	70.7	49.0	63.1	1.38	0.84
3	26.2	83.4	52.4	61.9	1.58	0.88
4	15.9	91.7	57.1	61.0	1.92	0.92
5	11.9	95.0	62.5	60.8	2.39	0.93
6	7.9	97.8	71.4	60.4	3.59	0.94
7	5.6	98.9	77.8	60.1	5.03	0.95
More than 7	4.8	99.4	85.7	60.0	8.62	0.96

## Discussion

This study of 307 fellowship bound internal medicine residents is the first to examine whether publication records at the time of fellowship match predict late publishing (between 3 and 7 years after fellowship match). With an area under the curve of 0.59, application publications fail as a predictor of late publications. We provide no evidence to support the widely held conception that those who publish at the time of fellowship match are more likely to publish in the long term. Additionally, our results are aligned with a small study of neurosurgical residents, which shows that publications do not predict academic or private practice careers [9].

The results presented here appear to contradict previous reports, which noted a strong, and statistically significant relationship between publications during residency and those afterwards [3] and publications during residency and choice of career (academic vs. private practice) [10]. Notably, the r-squared value we observed: 0.005 suggests that only one-half of one percent of variation in post-match publications is explained by application publications. However, simple comparisons may differ from more rigorous testing. For instance, although we noted that Late Publishers had more articles at time of application than Non Publishers (2.03 vs. 1.19), correlation coefficients and test characteristics, two more rigorous tests, were not met. Other groups have shown unprofessional comments on dean's letters are significantly associated with future board disciplinary action [11], however, more rigorous analyses demonstrate that there is no way to leverage this information as a useful screening tool, and it is far more likely to wrongfully cast doubt upon students [5]. Similarly, the publication record of an applicant at the time of fellowship match may lead fellowship directors to believe they have an understanding of that person's academic potential; however, our data do not show how this can be operationalized.

There are several limitations to our current work. The publication habits of the residents in the schools we examined may not reflect those of other schools. As with any study of this kind, the set of programs we examined is arbitrary. We chose major residency programs affiliated with top 20 medical schools according to US News and World Report, similar to the methodology used by Yang, et al. in their survey of urology publications [3]. We were unable to study the quality of publications. Perhaps residents who published more first author papers, or in journals of higher impact factor were indeed more likely to publish in the long term than those who did not. Additionally, we did not analyze whether the type of publication offered predictive power, i.e. do basic science publications predict future publication? Although, we could perform such an analysis, it would run the risk of generating false positives simply by virtue of multiple hypothesis testing [12]. We were not able to identify which residents earned other degrees (e.g. PhD), and whether those residents had different patterns of publication. As with any project of this kind, it is possible that any particular article may have been misattributed to a given author. In general, we erred on the side of inclusion when assessing publication histories, perhaps overestimating publication records. At the same time, there are reasons to hypothesize that we underestimated scholarship output. For instance, we did not consider other potentially worthy measures, such publications of book chapters, evidence updates, or non-peer reviewed medical newspapers. Surveys of residents may be best to ascertain these publications, and serve as a springboard for future research.

Finally, this project utilized the judgment of multiple different physicians; it is possible that others may disagree with our classification of articles. However, as articles were only classified based on the methods used, we doubt others would differ significantly. Despite these limitations, internal checks were used to detect misattributed articles, and we believe the results presented here are accurate for the group and time examined.

In addition to our findings, we suggest that our methodology of evaluating an association be adopted in future medical education publications. If groups identify factors that predict future performance, it would be useful to formally provide test characteristics of that factor, and not merely report its statistical significance in aggregate. Both in our findings and methods, our results may be of interest to a large group of fellowship and residency program directors as well as fellowship applicants.
